# Formation of Fused Images of the Land Surface from Radar and Optical Images in Spatially Distributed On-Board Operational Monitoring Systems

**DOI:** 10.3390/jimaging7120251

**Published:** 2021-11-25

**Authors:** Vadim A. Nenashev, Igor G. Khanykov

**Affiliations:** 1 Laboratory of Intelligent Technologies and Modelling of Complex Systems, Institute of Computing Systems and Programming, Saint Petersburg State University of Aerospace Instrumentation, 67 B. Morskaia St., 190000 Saint Petersburg, Russia; 2 Laboratory of Big Data Technologies for Sociocyberphysical Systems, St. Petersburg Federal Research Center of the Russian Academy of Sciences, 14 Line V. O. 39, 199178 Saint Petersburg, Russia; igk@iias.spb.su

**Keywords:** image segmentation, pixel clustering, fused image, small-sized on-board systems, contour points, location information, unmanned aerial vehicle, spatially distributed systems

## Abstract

This paper considers the issues of image fusion in a spatially distributed small-size on-board location system for operational monitoring. The purpose of this research is to develop a new method for the formation of fused images of the land surface based on data obtained from optical and radar devices operated from two-position spatially distributed systems of small aircraft, including unmanned aerial vehicles. The advantages of the method for integrating information from radar and optical information-measuring systems are justified. The combined approach allows removing the limitations of each separate system. The practicality of choosing the integration of information from several widely used variants of heterogeneous sources is shown. An iterative approach is used in the method for combining multi-angle location images. This approach improves the quality of synthesis and increases the accuracy of integration, as well as improves the information content and reliability of the final fused image by using the pixel clustering algorithm, which produces many partitions into clusters. The search for reference points on isolated contours is carried out on a pair of left and right images of the docked image from the selected partition. For these reference points, a functional transformation is determined. Having applied it to the original multi-angle heterogeneous images, the degree of correlation of the fused image is assessed. Both the position of the reference points of the contour and the desired functional transformation itself are refined until the quality assessment of the fusion becomes acceptable. The type of functional transformation is selected based on clustered images and then applied to the original multi-angle heterogeneous images. This process is repeated for clustered images with greater granularity in case if quality assessment of the fusion is considered to be poor. At each iteration, there is a search for pairs of points of the contour of the isolated areas. Areas are isolated with the use of two image segmentation methods. Experiments on the formation of fused images are presented. The result of the research is the proposed method for integrating information obtained from a two-position airborne small-sized radar system and an optical location system. The implemented method can improve the information content, quality, and reliability of the finally established fused image of the land surface.

## 1. Introduction

Nowadays, there is a growing interest in the research and development of two-position location-based on-board systems for land surface monitoring, especially for hard-to-reach places, prompt notification of natural and man-made emergencies, and other environmental disasters. These systems are based on small unmanned aerial vehicles (UAVs). The use of such systems makes it possible to achieve higher tactical and technical characteristics in comparison with single-position systems [1,2,3,4].

A review of single-position radar modes has shown the significant complexity of practical application of the known methods of terrain scanning [5,6]. In particular, use the entire list of conditions presents itself to be problematic when designing operational monitoring systems in a single-position version. The following functioning conditions of such systems should be highlighted:

- the possibility of search time minimization when observing zones, with the subsequent escort of the detected above-ground and ground objects on them, including people in trouble;
- increasing the resolution both in the “range” coordinate and in the azimuth coordinate in the quasi-real-time mode;
- provision of a low fixed false alarm probability when detecting physical objects of interest.

The third requirement, in turn, imposes the condition for the separation of signal, re-reflected from the land surface, against the background of noise and interference. In this case, the signal/background noise ratio should be as high as possible [7,8,9]. The conditions outlined above form the task of improving the existing modes of radar monitoring of the land surface.

In turn, application of a two-position version of the on-board monitoring system enables to reduce the review time, which is the main requirement in solving problems of operational monitoring of the land surface [5,6,10]. To meet the first and third requirements, actually it is advised to use the forward view to search for objects along the course of movement of spatially distributed airborne radars, as described in [5,6,9]. The forward view approach minimizes the time spent searching for areas and objects, crucial in operational search and rescue operations.

When implementing a forward observation along the course of on-board radar systems of a two-position system, it is important to consider, that its trajectory can be corrected upon approach before the observed area will be treated as an emergency zone or an environmental disaster zone. This aspect allows for time-savings when approaching the area under observation, compared to the mapping methods in the side-view mode, which would require a turn action, which would require additional time for exploration. In addition, with the implementation of such an observation mode of a two-position system and with the possibility of correcting the UAV’s movement when approaching the emergency area, the distance to it decreases. As a result of a decrease in the range, the signal/background ratio increases, hence, the third condition is satisfied.

The condition to increase the resolution in the azimuthal coordinate in real-time mode, obtained through the implementation of a two-positional version of the land surface observation in the forward viewing zone along the direction of movement of UAVs, is also the main performance characteristic when performing operational monitoring tasks. A similar requirement for the “range” coordinate got through the compression characteristics of the frequency or phase-modulated radar echo signal [2,7,8]. Thus, the advantage of small-sized spatially distributed airborne radar systems in terms of the implementation of operational monitoring systems of the terrain is determined.

However, the radar data received from the UAV aircraft does not always reflect the whole location setting in the observed area of the terrain. It is hard to solve the problems of zone classification, object recognition, boundary detection of small objects, through radar means [10]. In this regard, tasks of improvement of quality, accuracy, and information content of displaying relevant information about the land surface are especially important. One of the options for a qualitative increase in the information content of the land surface is to use additional location sources of other physical nature [10,11,12,13] in combination with radar facilities.

For these reasons, the development of multi-sensor and multi-position onboard location systems, as well as operational monitoring methods on their basis becomes especially urgent today.

Currently, there are several frequently used sources of location information using multiple kinds of natural reflections. At the same time, none of these sources is unique and suitable for displaying of the entire spectrum of the required integral picture of the location situation behind the observed area.

In journals [14,15], presented to compare the main characteristics of sources of heterogeneous location information, namely radar, optical, laser, and ultrasonic. Among the main characteristics of sources of location information are the following ones:

- distance to the object;
- the ability to detect objects in the near and far zones;
- the viewing angle;
- the ability to determine the radial and tangential speed;
- the ability to classify objects of interest;
- the influence of illumination and weather conditions on the quality of the information received, as well as
- the ability to highlight the boundaries of objects of interest.

The result of studies [12,15] show that to fully satisfy the requirements for sources of location information, the most effective approach is the combined use of different sources. In this case the disadvantages of some sources are mitigated by the advantages of others. It follows from data, presented in [10,12,14,15], that an effective integration option is to combine information from radar and optical sources in a single entity. Such integration enriches the information content when displaying the location settings in the observed areas and relevant objects from the UAV boards, which includes the selection of detected objects, determination of their coordinates, selection of motions of physical objects, including people, as well as the implementation of high-precision mapping of the land surface with the ability of classification of both observed areas and objects on them.

In addition, new solutions have recently been developed [16,17] that allow optical images to get rid of their limitations, such as haze, fog, rain and snow droplets and other particles floating in the air and getting into the visibility range of the optical location system. These solutions mitigate the deterioration of visibility caused by bad weather conditions in real time. Thus, it once again emphasizes that it is advisable to choose the fusion of optical and radar images as the main sources of information.

Thus, it is noted that the mutual complementarity between radar and optical images carries significant potential for applications of remote sensing of the terrain.

There exist some methods of forming fused images of the terrain [18,19], upon analysis of which it should be concluded, that combining optical and radar images is more difficult, than simply combining two optical images, since the values of pixel brightness in grayscale on radar images do not adequately map to the values of pixel brightness on similar optical images. The fusion of remote images is carried out because of the fusion of measuring radar information (range, angle). The optical image does not contain this information, so a different approach is required. Fusion of optical and radar images is a more difficult process than a regular fusion of two optical or two radar images, since the values of pixel brightness in grayscale in radar images do not correlate with the values of pixel brightness in similar optical images. This is the significant difference in fusion of homogeneous and heterogeneous images.

Methods and strategies for the formation of fused images can be conditionally divided into three types [20]:

- aggregation at the pixel level;
- integration at the level of features;
- integration at the decision-making level.

The peculiarity of aggregation at the pixel level is the possibility of using data from a type of source of location information, which outweighs the disadvantages of the high computational complexity of an integrated system.

The second type of integration requires the construction of a system for the isolation of independent features, retrieved from each separate heterogeneous channel of information. The implementation of this type of integration requires less computational overheads compared to the first type, because the complex analysis of data from various sources of location information is not required.

The third type of integration is implemented for each of the heterogeneous channels on the basis of solutions determined separately according to information from the corresponding heterogeneous sources. Since useful information and decisions are determined independently in heterogeneous channels, this approach is less demanding on the data co-registration quality.

Integration at the decision-making level is based on decisions made separately according to data, acquired from each of the heterogeneous channels. Since data and decisions are made in each channel independently, this method is less sensitive to the quality of data co-registration. However, to ensure a higher quality of stacked complex images, the accuracy of data processing in each channel must improve. This level was characterized by the least computational complexity.

The ability to combine different types of data from heterogeneous sources of location information, independence from errors at the stage of registration of this data, and accurate methods of data combining can be mentioned as advantages of performing this task at the decision-making level, rather than at other levels. There is a large body of research on approaches to merging solutions for merging radar and optical data [21,22,23,24].

Fusion of radar and optical data from the relevant sources of location information with their inherent differences requires the development of new methods and strategies for the formation of complex images of this kind.

When fusing multi-angle images with a fixed number of reference points, the quality of final fused image is unpredictable in advance, and further improvement is not feasible even if the result is unsatisfactory. Therefore, in present research, a new fusion method is proposed, which, in comparison with the other existing ones, allows establishing a fused image of the terrain based on multi-angle heterogeneous images, using an adaptive (iterative) mechanism. This mechanism improves the quality of synthesis and increases the accuracy of integration, as well as the information content and reliability of the final fused image by using the pixel-clustering algorithm, which generates many partitions into clusters.

The original multi-angle images, taken by the onboard equipment of multi-positional location systems are docked into a single composite image and, using the pixel clustering algorithm, are reduced to several grayscale levels while retaining the characteristic boundaries. A particularity of the applied pixel clustering algorithm is the generation of a series of image partitions into clusters because of a variable number of clusters. This feature allows selecting the appropriate partition of pair of docked images from the generated series.

The search for reference points on isolated contours is performed on a pair of left and right parts of the docked image from the selected partition. For these reference points, a functional transformation is determined. Having applied it to the original multi-angle heterogeneous images, the degree of correlation of the fused image is assessed. Both the position of the reference points of the contour and the target functional transformation itself are refined until the quality assessment of the fusion becomes acceptable. The type of functional transformation is selected based on clustered images and then it is applied to the original multi-angle heterogeneous images. This process is repeated for clustered images with greater granularity in case if quality assessment of the fusion is considered to be poor.

The problem under consideration consists in the development of a new method for fusion and processing of heterogeneous data received from sources of location information based on different physical principles, that is, two-position radar systems using small-sized airborne radars and from optical-location systems based on controlled UAVs, to improve the alignment accuracy, information content, and reliability of representation of the location situation of the land surface. The method was developed to prove the concept of an effective and reliable display of the location situation formed by a two-position system in the forward viewing zones of the airborne radar and optical location system (OLS) while preserving all the important qualities of the original images that complement each other.

## 2. Approach to Fusion Multi-Angle Optical and Radar Images

As discussed above, it is difficult to use the optical and radar systems separately for the implementation from onboard systems for operational monitoring on the land surface. In this regard, it becomes necessary to integrate optical radar information received from multiple kinds of devices from the units of a spatially distributed UAV system.

**Figure 1:**
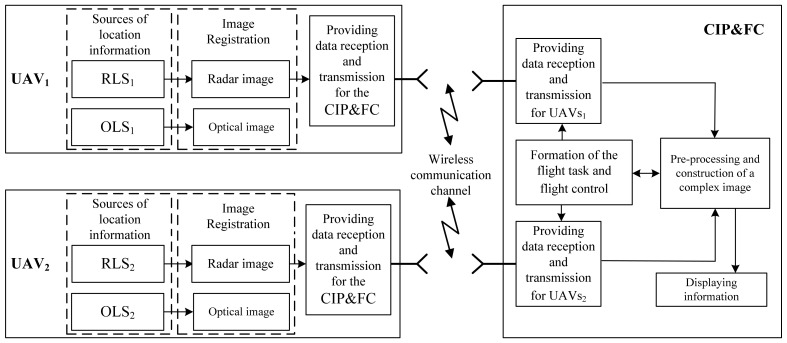
Block diagram of the radio-optical complex.

Figure 1 structurally depicts two UAVs, each of which is equipped with sources of location information included in a multi-position system. These sources are an airborne radar system (ARS), which performs mapping by synthesizing an antenna aperture, and an optical location system (OLS). The output of the operation of these heterogeneous location systems is high-resolution location images.

In the flowchart in Figure 1, the following components are the main blocks for the successful performance of the image fusion process in a radio-optical two-position complex are shown. These are a high-speed information exchange channel between the UAV equipment and the center for integrated processing and flight control (CIP&FC), systems for radar image fusion, and optical image fusion.

In this block diagram, great importance is attached to a high-speed communication channel, through which the images formed by the equipment of the on-board radar and the OLS are exchanged both among themselves and are transmitted to the CIP&FC of the UAV, since this data batch contains a fairly large amount of information. In addition, the development of a wireless channel for a high-speed multi-position information exchange system must include implementation of masking, compression, and coding algorithms generated by the UAV equipment of images [25,26,27,28,29].

The functional essence of the fused image formation in a two-position UAV system is as follows. Heterogeneous image of the observed area of the land surface are transmitted via a wireless communication channel from each location system from the equipment of the corresponding two-position location systems RLS_1_, RLS_2_, and OLS_1_, OLS_2_ of two UAV crafts to the CIP&FC UAV. The preliminary preparation of the incoming images is performed in CIP&FC. Next, a complex image has formed.

**Figure 2:**
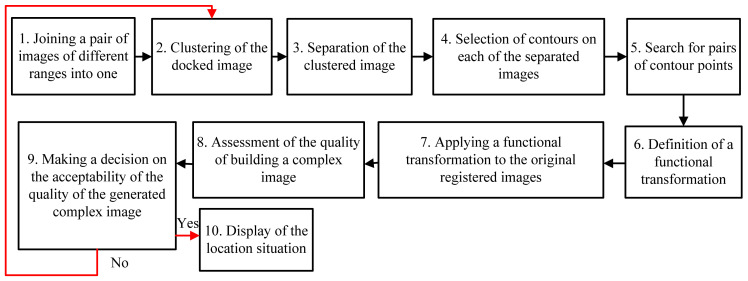
Scheme of implementation of the method for fused image formation.

Let us extensively consider the image fusion method. Its highlight is the use of an algorithm for high-speed clustering of image pixels formed by the equipment of the location devices of a multi-position UAV system. Figure 2 shows a flowchart of the implementation of the method for the fusion of multi-angle and heterogeneous images. The essence of each block is clear from its corresponding name.

Next, let us consider in detail the image segmentation algorithms that underpin the method for fusion of multi-angle and heterogeneous images of the land surface.

## 3. Image Segmentation and Border Isolation Methods

Segmentation involves image division into many different regions (clusters of pixels or image segments) in reference to standard features, for example, brightness, color, gradient value, pixel location. The segmentation task refers to the preliminary stage of image processing. In the chain of technological workflow, the next steps of isolation, object recognition, scene analysis, and situation prediction depend on the segmentation results. Segmentation is applied in many practical areas, such as healthcare, security systems, remote sensing of the Earth’s surface, and other applications such as area search, 3D modeling, visualization, and navigation. In general, segmentation techniques provide a compact representation of the data, such that all subsequent processing is performed at the region (cluster or segment) level rather than at the pixel level, thereby minimizing computation.

In [30], a classification scheme for segmentation algorithms is given, based on partitions number to an output of the algorithm. Here the segmentation algorithms are divided into categories that return either a single partition or a series of partitions to the output. Algorithms generating a set of partitions are of interest since one or several suitable partitions can always be selected from a series. For example, [31,32] methods allow bypassing the problems of under-segmentation and over-segmentation due to the variable number of clusters being considered.

The clustering of color images is not implemented due to the high computational complexity, which increases quadratically with the increase in the number of pixels within the scope of this research. The clustering of grayscale images is applied. The latter approach is implemented through multi-threshold processing [31], which sequentially enlarges the adjacent groups of pixels in the brightness histogram.

There is no single generally accepted segmentation algorithm. For a specific task, either a custom segmentation algorithm is developed, or several existing methods are selected. In the present work, two image segmentation algorithms are applied, each of which fulfills a specific task in the application of the image fusion method. For example, using the multi-threshold processing algorithm [31], a series of piecewise-constant partitions of varying degrees of detail into characteristic regions are generated. Each partition in the series enables the granularity of the captured scene to varying degrees. To get a series, one or several suitable partitions are selected, on which the corresponding operation is performed using the Canny edge detector [33]. This set of segmentation algorithms avoids the previously mentioned over-segmentation and under-segmentation problems [34].

### 3.1. Multi-Threshold Processing

The idea of the multi-threshold method [31] used in this research is to sequentially combine pairs of adjacent clusters on the brightness histogram. Initially, one column of the brightness histogram corresponds to one cluster. All adjacent pairs of clusters are inspected in one pass along the brightness histogram. The distance function is calculated for each pair of clusters. At the end of each pass along the brightness histogram, a pair of adjacent clusters with the minimum value of the distance function is combined. Each cluster is characterized by the number of pixels in it and by the average brightness value.

In the original multi-threshold processing method [31], the function of the distances between pairs of adjacent clusters calculated through the product of the intraclass and interclass variances. The brightness histogram is considered as a function of the probability density, which involves massive cumbersome calculations. In a modified version of the multi-threshold processing method, a function of the distance between adjacent pairs of clusters is the increment in the total squared error Δ*E*:

(1) $$\Delta E=\frac{n_{1}n_{2}}{n_{1}+n_{2}}\left|I_{1}-I_{2}\right|,$$

where *n*_1_ and *n*_2_—number of pixels in clusters 1 and 2, *I*_1_ and *I*_2_—average brightness of pixel clusters 1 and 2. Δ*E* takes a minimum value of 0 when parameters *I*_1_ and *I*_2_ are equal for any area of clusters 1 and 2. Δ*E* takes on a maximum value of 127.5 when the brightness of clusters 1 and 2 take opposite values of 0 and 255 and, at the same time, the areas of clusters 1 and 2 are unit ones: *n*_1_ = 1, *n*_2_ = 1.

Replacement of the distance function is reasonable for several reasons. First, the use of the increment in the total squared error Δ*E* (1) halves the volume of accompanying operations. Second, quality indicator of the current piecewise constant partitioning of the image into clusters is the accumulated value of the total squared error *E*, which is calculated through the value of the indicator of the previous partition:

(2) $$E_{step\;i}=E_{step\;i-1}+\Delta E_{step\;i},\;\;\;\;E_{step\;0}=0.$$

Thirdly, the application of the increment of the total squared error Δ*E* (1) entails a change in the order of the sequence of combining clusters and, hence, the calculation of other average values for the clusters. It is established, that the new sequence values of the total square error, characterizing the quality of the partitioning into clusters, form a convex curve throughout.

The total squared error *E* is related to the standard deviation *σ* by the equality:

(3) $$\;\sigma =\sqrt{E/M},$$

where *M*—total number of pixels in image. Both values unambiguously characterize the quality of the partition. However, the first one (Equation (2)) is convenient to use in calculations, and the second one (Equation (3)) is convenient to display the results.

The original method [31] and its modification are applicable only for grayscale images. In the general case, all partitions into clusters are generated within 256 passes over the brightness histogram. In the particular case, when the number of non-empty gray levels is equal to *K* < 256 and it is required to find a partition into *t* < *K* gray levels, (*K*−*t*) passes over the brightness histogram are required.

### 3.2. Canny Edge Detector

Boundaries of previously isolated areas can be detected using several filters. The simplest are convolution filters, for example, Sobel, Prewett, Roberts, Sharr, etc. There is no fundamental technological difference in the implementation of convolution filters. The changes are applied to the convolution kernel only. Convolution filters have the advantage of being easy to implement. However, highlighting a false border is a significant drawback.

The disadvantages of convolution filters are overcome by the Canny edge detector [33], whose algorithm consists of five stages: smoothing, gradient detection, suppression of “non-maximum” double threshold filtering, and boundary refinement. The first two stages are generic. Smoothing can be performed not only with a Gaussian filter but also, with a median filter. The situation is similar to the gradient search stage, which used various convolution operators.

#### 3.2.1. Smoothing Stage

Smoothing is performed by a Gaussian filter size of (2*k* + 1) × (2*k* + 1), where *k* is the parameter of the side length of the filter kernel. Smoothing is necessary to avoid false edges.

Тhe standard deviation σ must be specified to create a Gaussian filter kernel. The larger is the σ value, the smoother is the target image. Elements *h*_(*i, j*)_ of the Gaussian filter kernel with coordinates (*i*, *j*) are determined by the formula:

(4) $$\begin{array}{c} h_{\left(i,j\right)}=\frac{1}{2\pi \sigma^{2}}exp\left(-\frac{{\left(i\;-\;\left(k\;+\;1\right)\right)}^{2}\;+\;{\left(j\;-\;\left(k\;+\;1\right)\right)}^{2}}{2\sigma^{2}}\right),\;\\ \left(i,j\right)\;\in \;\left[1;2k+1\right]. \end{array}$$

The size of the Gaussian filter kernel affects the performance of the Canny edge detector. The larger it is, the lower is the sensitivity of the Canny edge detector to noise. But at the same time, with an increase in the kernel size, the error in the localization of the edges increases insignificantly. Practice shows that kernel size of 5 × 5 (*k* = 2) is sufficient for most cases.

#### 3.2.2. Gradient Search Stage

The search for the gradient is carried out using the convolution of the original image A with integer Sobel filters in the vertical and horizontal directions. Boundaries are marked where the gradient of the image is at its maximum value.

The horizontal *G_x_* and vertical *G_y_* gradients are found as follows:

(5) $$G_{x}=\left[\begin{array}{ccc} 1 & 0 & -1\\ 2 & 0 & -2\\ 1 & 0 & -1 \end{array}\right]\times A,\;G_{y}=\left[\begin{array}{ccc} 1 & 2 & 1\\ 0 & 0 & 0\\ -1 & -2 & -1 \end{array}\right]\times A.$$

The modulus of the gradient vector is determined through the components of the gradient (*G_x_*, *G_y_*) as follows:

(6) $$\left|\nabla G\right|=\sqrt{G_{x}^{2}+G_{y}^{2}}.$$

The direction of the gradient vector is defined as follows:

(7) $$\alpha \left(x,y\right)=arctg\left(\frac{G_{y}}{G_{x}}\right),$$

where $\alpha \left(x,y\right)$ is the angle between the direction at the point (*x*, *y*) and the O*x* axis. The tilt angle is rounded to the nearest 0, 45, 90, or 135 degrees.

#### 3.2.3. Stage of “Non-Maximum” Suppression

In [34], the concept of “non-maximum” is introduced. The image pixels, in which the maximum value of the local gradient in the direction reached, are called “maximums”. All other pixels, in the local maximum of the gradient in which the direction is not reached, are recognized as “non-maximum”. Pixels—“maximum” form boundaries preserved. “Non-maximum” values are suppressed. Since there are four directions of gradient change, each case has its condition for recognizing a pixel as a “maximum”:

(a) When the angle α of the direction of the gradient is 0° or 180°, a pixel is considered to be a boundary-forming “maximum” if its intensity is greater than that of pixels located above or below the considered one: $\nabla G\left(x-1,y\right)<\nabla G\left(x,y\right)>\nabla G\left(x+1,y\right)$;
(b) When the angle α of the direction of the gradient is 90° or 270°, a pixel is considered to be a boundary-forming “maximum” if its intensity is greater than that of pixels located to the left or to the right of the considered one: $\nabla G\left(x,y-1\right)<\nabla G\left(x,y\right)>\nabla G\left(x,y+1\right)$;
(c) When the angle α of the direction of the gradient is 45° or 225°, a pixel is considered a boundary-forming “maximum” if its intensity is greater than that of pixels located in the upper right and lower left corners of the considered one: $\nabla G\left(x-1,y+1\right)<\nabla G\left(x,y\right)>\nabla G\left(x+1,y-1\right)$;
(d) When the angle α of the direction of the gradient is 45° or 225°, a pixel is considered to be a boundary-forming “maximum” if its intensity is greater than that of pixels located in the upper left and lower right corners relative to the considered one: $\nabla G\left(x+1,y+1\right)<\nabla G\left(x,y\right)>\nabla G\left(x-1,y-1\right)$.

#### 3.2.4. Stage of Double Threshold Filtering

The Canny edge detector uses double threshold filtering to keep the “strong” data, discard the “false” data, and check for “weak” boundaries in the next stage. The “strong” are those boundaries, whose pixel brightness values are above the upper set filtering threshold. Pixel brightness values of “false” boundaries are below the lower set threshold. If the pixel brightness falls within the range between the upper and lower set thresholds, such a pixel belongs to the “weak” border.

Depending on the set values of the threshold levels, the images of the boundaries at the output of the Canny detector differ. At low thresholds, most of the boundaries will be preserved, but the resulting image may be overly segmented. Conversely, at high threshold values, pixels referred to as “weak” boundaries are lowered, and the boundary portrait may be fragmented.

#### 3.2.5. Stage of Boundary Refinement

The final stage corrects the double threshold filtering stage by revising the pixels referred to as “weak” boundaries. The pixels of the “weak” borders are assigned to the pixels of the “strong” ones when they touch in one of the eight directions with the pixels of the “strong” border. Otherwise, pixels of “weak” edges are suppressed.

## 4. Experiments on Fusion of Multi-Angle Images

**Figure 3:**
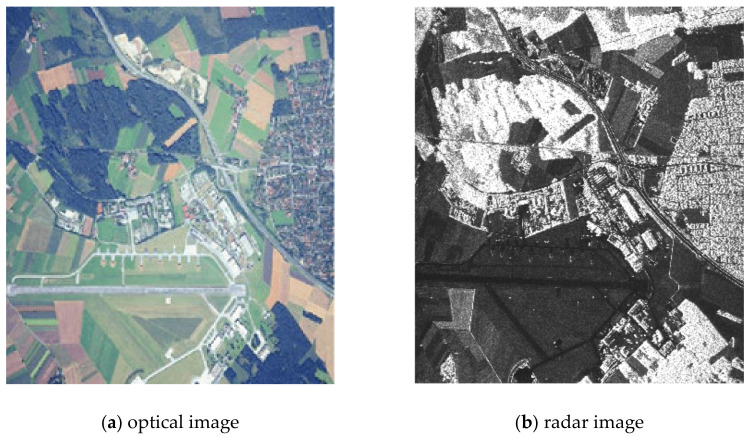
Optical and radar images of the same area.

The source location images of the land surface, formed by the onboard equipment of multi-position location systems, are multi-angle and heterogeneous ones (Figure 3a,b). The fusion method includes the operation from joint pre-processing of a pair of original images docked into a single composite image. The pixel clustering algorithm [31] enabled to reduce several gray levels in the image, allowed the selection of some areas while preserving their boundaries. Furthermore, the isolation of borders on the clustered image is done by an appropriate filter.

The method of fusion multi-angle images, considered in this research, is based on pairs of contour points and pairs of clusterized images. The workflow for joint clustering of pixels implies that two original images are pre-docked (concatenated) into a single image. The clustering allows for the selection of the same areas under different angles in a similar way. The clustering procedure used in the present research generates a series of piecewise constant partitions of the target image, transformed into pixel clusters. From the generated series of partitions, a suitable partition is selected, which is back-divided into left and right clustered multi-angle images. Selection of the partition begins from the smallest number of clusters, progressively increasing the granularity of the selection. At the same time, at the boundaries of the previously isolated areas on the separated pair of images (left and right clustered images), the search for control points is performed. Based on the selected pairs of control points, a functional transformation is established. Then it is applied to the original pair of multi-angle images. The search for functional transformation is repeated until the decent quality of the fusion is achieved. A gradual increase in the pair’s granularity of clustered images produces new boundaries and causes the definition of new pairs of reference points on them.

**Figure 4:**
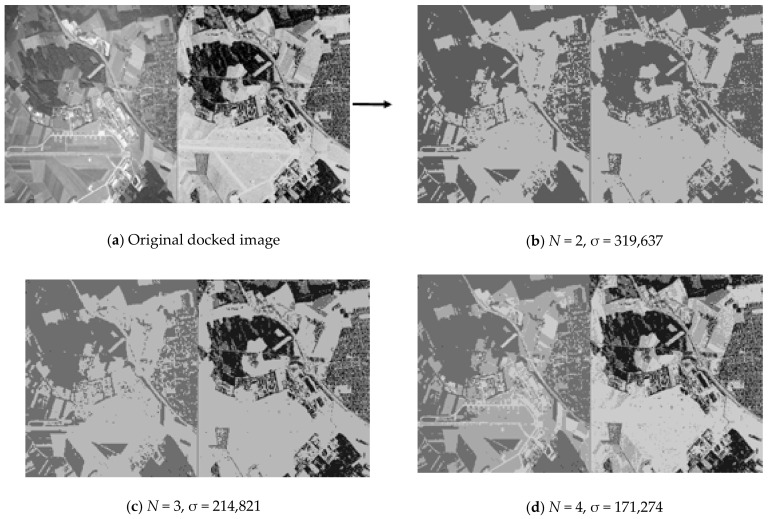
Part of a series of partitions of the docked optical and radar images into pixel clusters by means of multi-threshold processing.

**Figure 5:**
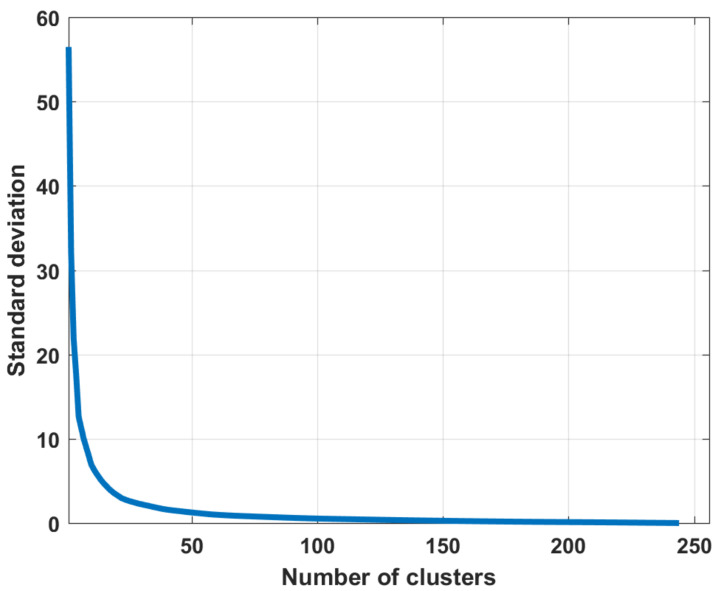
The dependence of the values of the standard deviations σ on the number of clusters in the partition.

**Figure 6:**
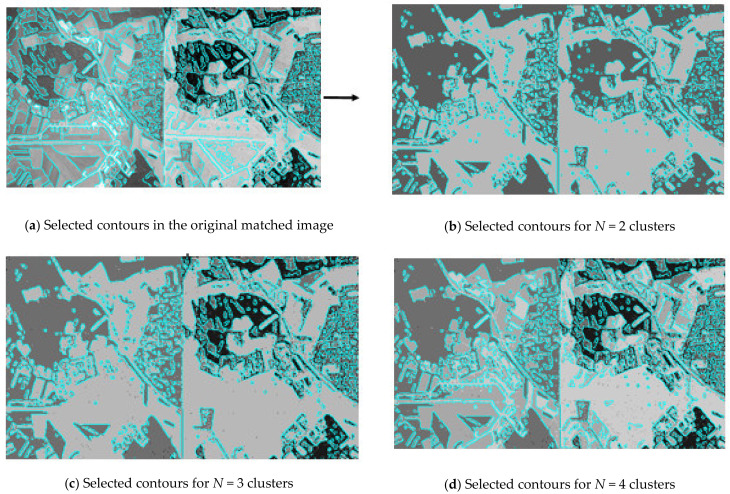
Boundary isolation by Canny edge detector on piecewise-constant partitions.

The first steps of the fusion method (see Figure 2) of multi-angle heterogeneous images are presented in Figure 4, Figure 5 and Figure 6. Figure 4 shows part of the results of clustering a composite image, namely, partitions into 2, 3, 4 clusters from a full set of 256 clusters. Next, Figure 6 shows the results of implementation of Canny edge detector.

### 4.1. Example of Multilevel Threshold Processing

Figure 4 shows part of the sequence of partitions of the original optical and radar images into clusters by the multilevel threshold processing method [31]. In order to isolate similar areas in different images in a similar way, in present research, the technique of joint processing of two images is used. The original docked image is shown in the upper left corner. It is obtained from the original optical image of the land surface converted into grayscale and a color-inverted radar image of the same area. Conversion to grayscale and color inversion enabled to bring identical objects in dissimilar images to the same shades of gray. To the right of the original docked image is a part of the sequence of partitions into clusters. Each partition is labeled with the number of clusters into which all pixels of the original image are divided and contains the value of the standard deviation σ characterizing the quality of the partition. The lower is the value of σ for the same number of clusters, the better is the quality of the partition. It is clearly seen that the same objects are isolated in a similar way already on docked partition into two clusters (*N* = 2). A further increase in the number of clusters details the internal structure of the caught-on-image objects.

For the original docked image shown in Figure 4, 256 piecewise-constant partitions are available. Figure 5 shows the standard deviation σ curve corresponding to the entire sequence of piecewise-constant partitions generated by the upgraded multi-threshold processing algorithm.

### 4.2. Example of Border Isolation with the Canny Edge Detector

Figure 6 demonstrates the border isolation by the Canny edge detector on the original docked image and on a part of a series of partitions into clusters. The boundaries were isolated using the R2020b MATLAB software tooling (MathWork, Natick, Apple Hill Campus, Massachusetts, MA, United States) in automatic mode. The smoothing parameter σ is equal to $\sqrt{2}$. The lower filtration threshold is 0.01, the upper one is 0.1. Similar to Figure 4, the original docked image is located in the upper left corner of Figure 6. Low values of the filter thresholds highlighted the smallest differences in brightness. The border image turned out to be oversaturated. Partitions, containing 2, 3 and 4 clusters are located next to it. The boundaries isolated by the Canny edge detector on piecewise-constant partitions and previously generated by the multi-threshold processing method are highlighted in turquoise. Notice that the length of the border increases with the greater granularity of each individual partition.

Preliminary use of pixel clustering generates image partition into clusters, merging piecewise-constant areas and, accordingly, excluding borders. After that convolution filters isolate left borders in image partition. There is no principal difference between the convolutional filters used here. However, Canny edge detector was implemented in present research. It requires specification of values of several parameters (smoothing, lower threshold, upper threshold). The smoothing parameter equal to $\sqrt{2}$, lower threshold equal to 0.01, upper threshold equal to 0.1 were selected empirically.

**Figure 7:**
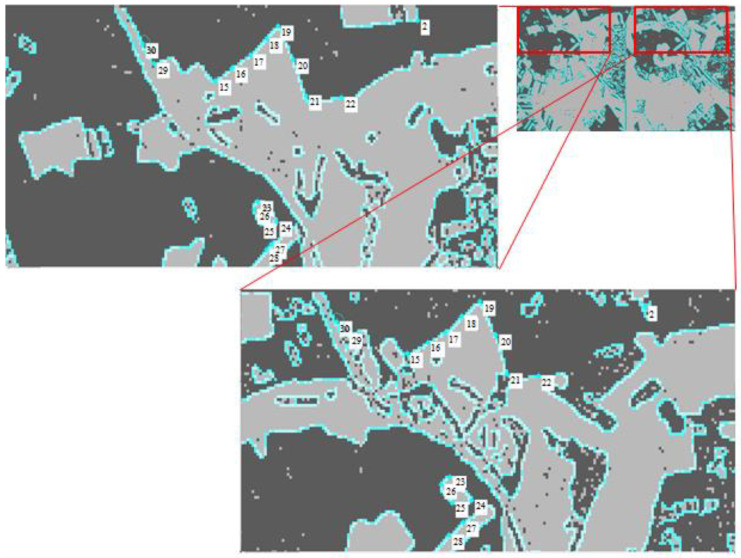
Search for pairs of reference points of contour on a clustered pair of multi-angle images.

Further, it is necessary to find pairs of contour points on pairs of clustered images with a highlighted border executing block 5 of the method presented in Figure 2. Figure 7 shows enlarged fragments of the same section of the observed zone on a partition with a isolated border after clustering images (*N*cl = 2). Pairs of control points similar in relative position to each other on the contour were found on the isolated boundaries [35].

After the pairs of reference points on the contours are found, a functional transformation is fitted from them (block 6 of Figure 2), which is applied to the original images (block 7 of Figure 2).

**Figure 8:**
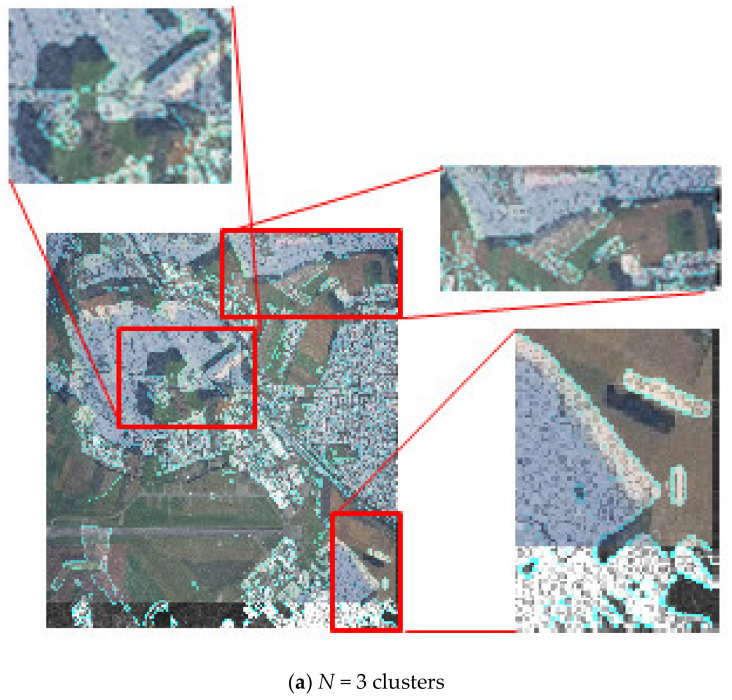

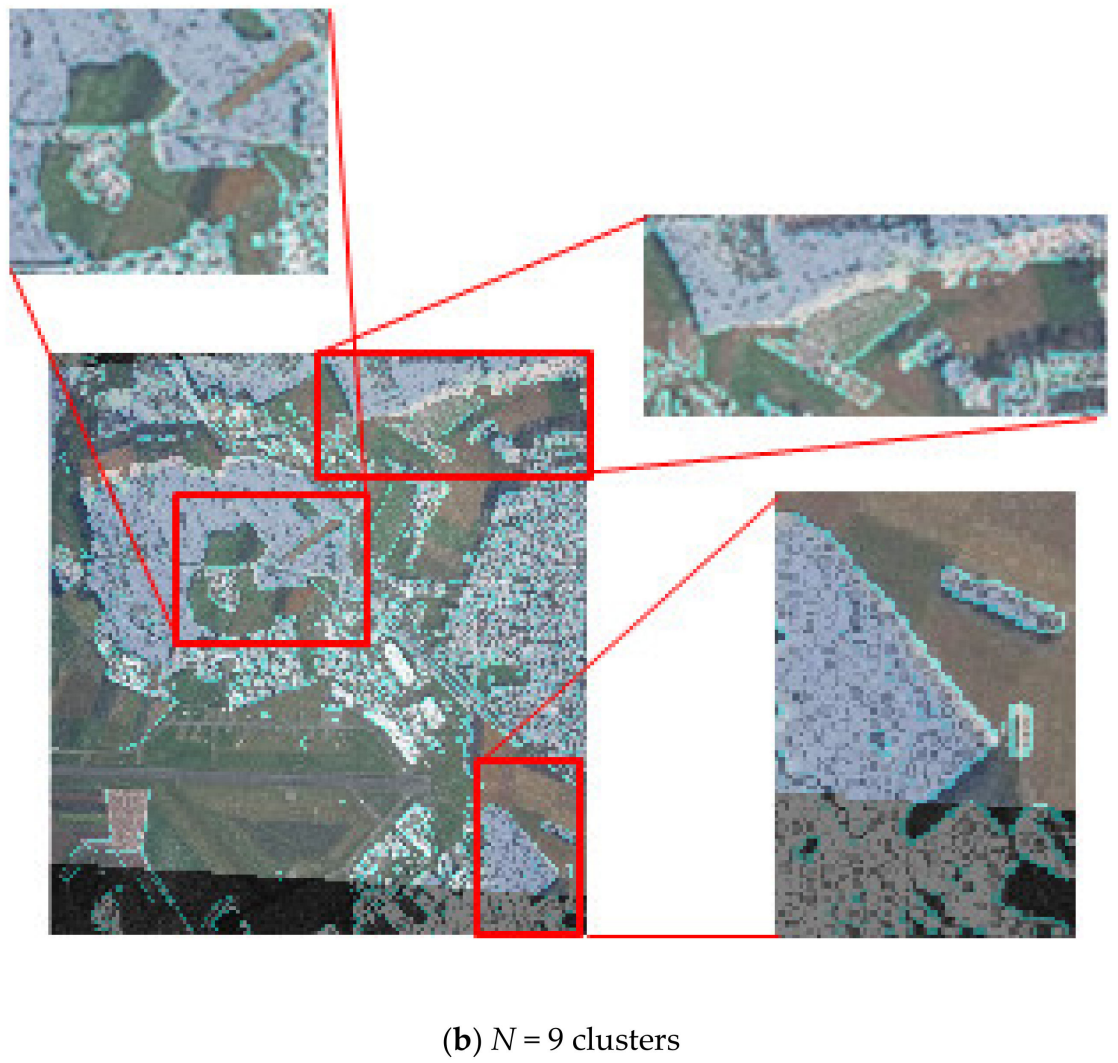
Assessment of the quality of the results of fusion of the original pairs of images with a different number of clusters and, the respective reference points in the partition.

The eighth block of the method (see Figure 2) evaluates the quality of fusion of multi-angle heterogeneous images and makes a decision on the acceptability of the quality of fusion. Figure 8 depict the quality assessment step. The result of Figure 8 can be regarded as unsatisfactory due to the significant discrepancy between the optical and radar layers for *N*cl = 3. If the result of the quality assessment is unsatisfactory, you should return to block 2 (see Figure 2) and choose a partition with a large number of clusters. Figure 8b shows a satisfactory result. The layers match almost completely.

To assess the fusion quality, it is necessary to use the similarity function, which allows assessing the degree of similarity of the contours of the characteristic areas of two different images when establishing a fused image. For this, the calculation of the two-dimensional cross-correlation function [36] of the contours of the clustered images is used. An ideal two-dimensional normalized correlation function is characterized by a single peak of small width at the origin. And outside this peak its values are equal to zero, which is achieved in complete matching of the image contours. Obviously, in practice, the complete matching of the contours of two different images practically cannot satisfy these properties, with the exception of rare cases of combining simple landscapes of the terrain, due to the effect of noise and distortions in the operational modes of onboard sources for generating of location information.

**Figure 9:**
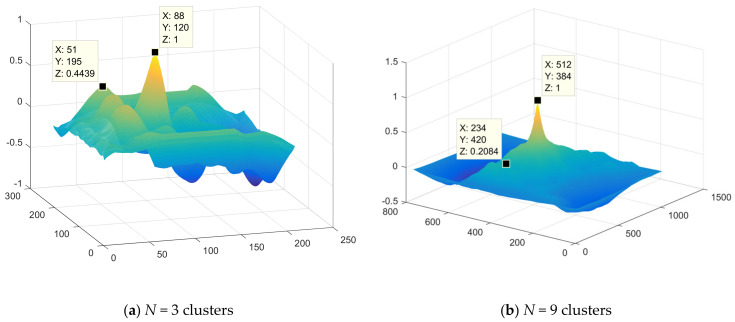
Plot of two-dimensional correlation function.

Figure 9 shows a plot of the two-dimensional correlation function of the contours with *N*cl = 3 and *N*cl = 9, respectively.

The acceptability of fusion accuracy value when implementing this method of fused image establishment can be determined by the maximum modulus of the side lobes (MSL) of the normalized two-dimensional correlation function.

In this case, for example, for *N*cl = 3, the considered MSL turned out to be equal to 0.4439, and the root-mean-square error of contour matching is ±6–7 pixels. With Ncl = 9, MSL turned out to be equal to 0.2084, and the root-mean-square error was ±3–4 pixels, which corresponds to an acceptable estimate of the comparison error.

**Figure 10:**
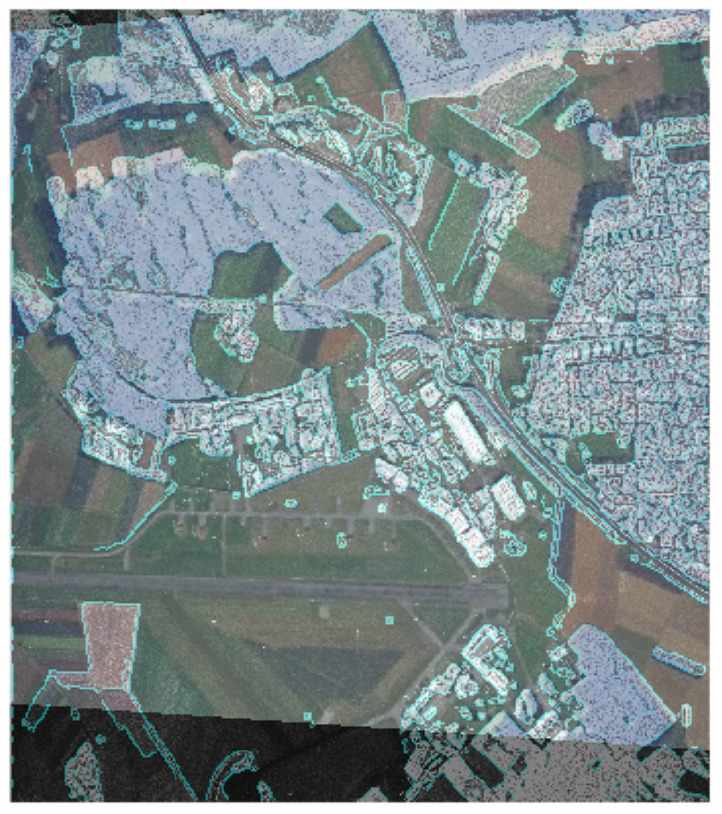
The result of the fusion of optical and radar images.

Figure 10 shows the result of the establishment of a fused two-layer image based on combining the original images (optical and radar) obtained from the on-board location devices of the multi-position system.

In Figure 10, the original heterogeneous images are fused into a single complex image by selecting the most important various components from both the original images. The contours of the characteristic areas are highlighted in turquoise in Figure 10. The obtained fused image contains two layers and has an optical and a radar layer. In this case, two heterogeneous layers mutually complement each other and, depending on the goals of onboard monitoring, each of the layers can be enhanced or weakened. Figure 10 illustrates the case where the optical layer above the radar is more enhanced.

**Figure 11:**
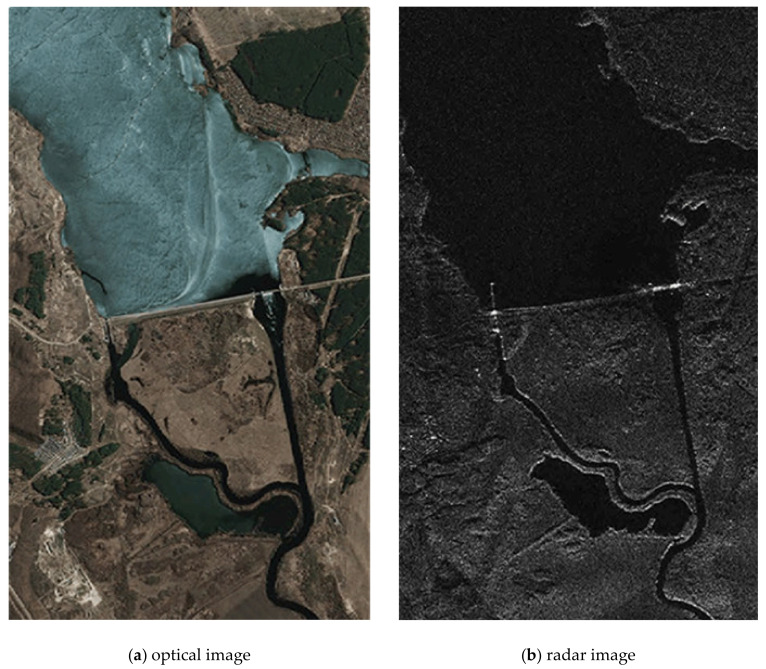
Heterogeneous images of the terrain.

**Figure 12:**
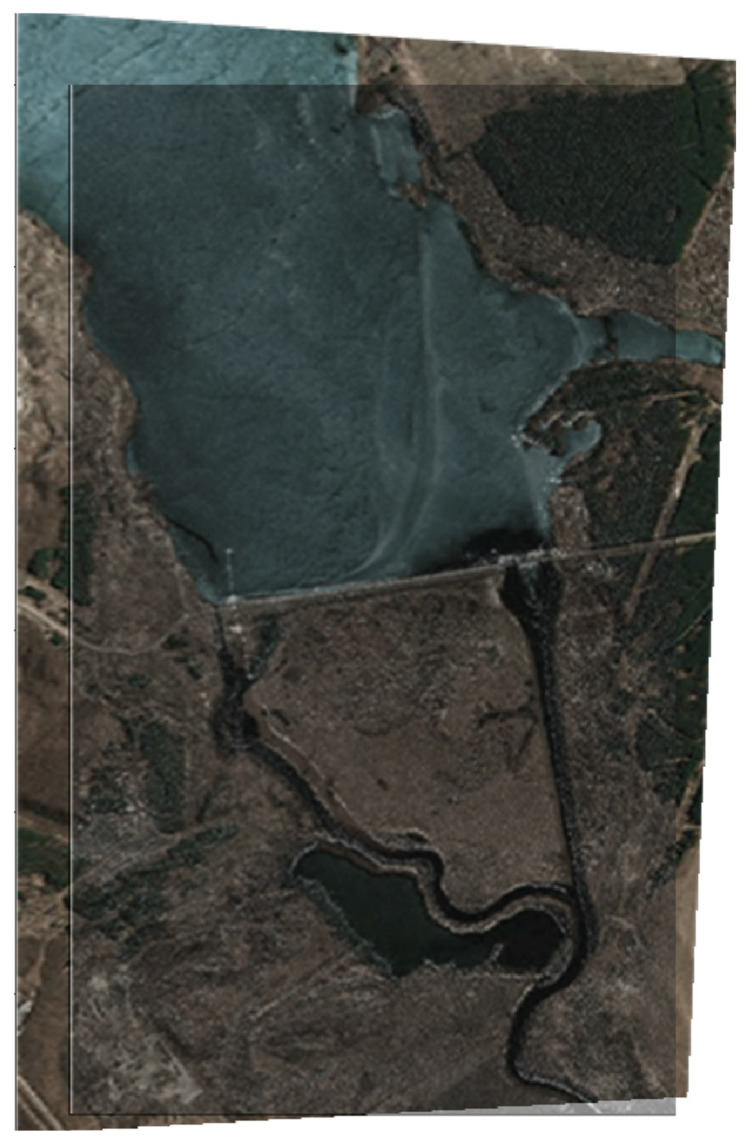
Fused image.

A similar experiment was performed for a pair of heterogeneous images shown in Figure 11 (see Figure 12).

It is sensible to use the first case when clarifying the classification of the observed areas, as well as ground and above ground objects. The second case is applicable for the initial detection of objects in difficult weather conditions and seasonal visibility conditions, as well as in conditions of destructive effects on the radar information channel. At the same time, with different contrasts of heterogeneous images of the land surface applied, structural changes in the landscape can be clearly distinguished when the digital map of the area is updated.

**Figure 13:**
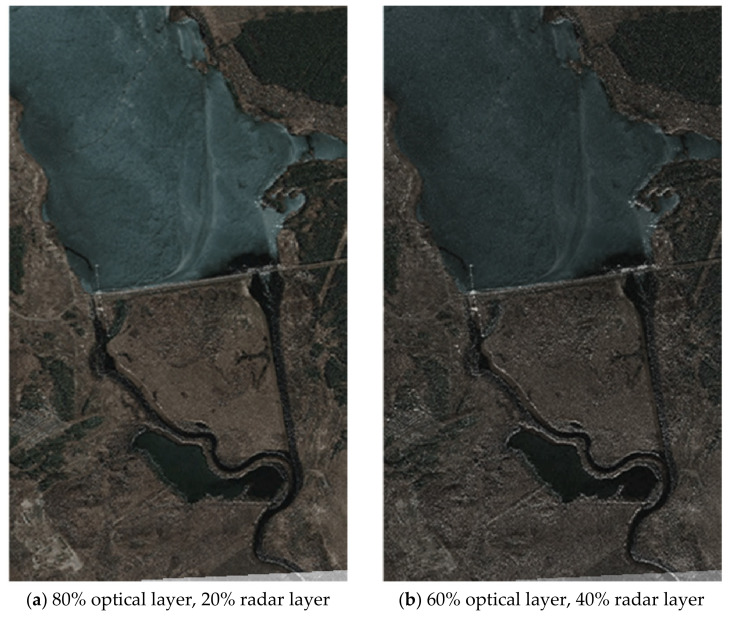

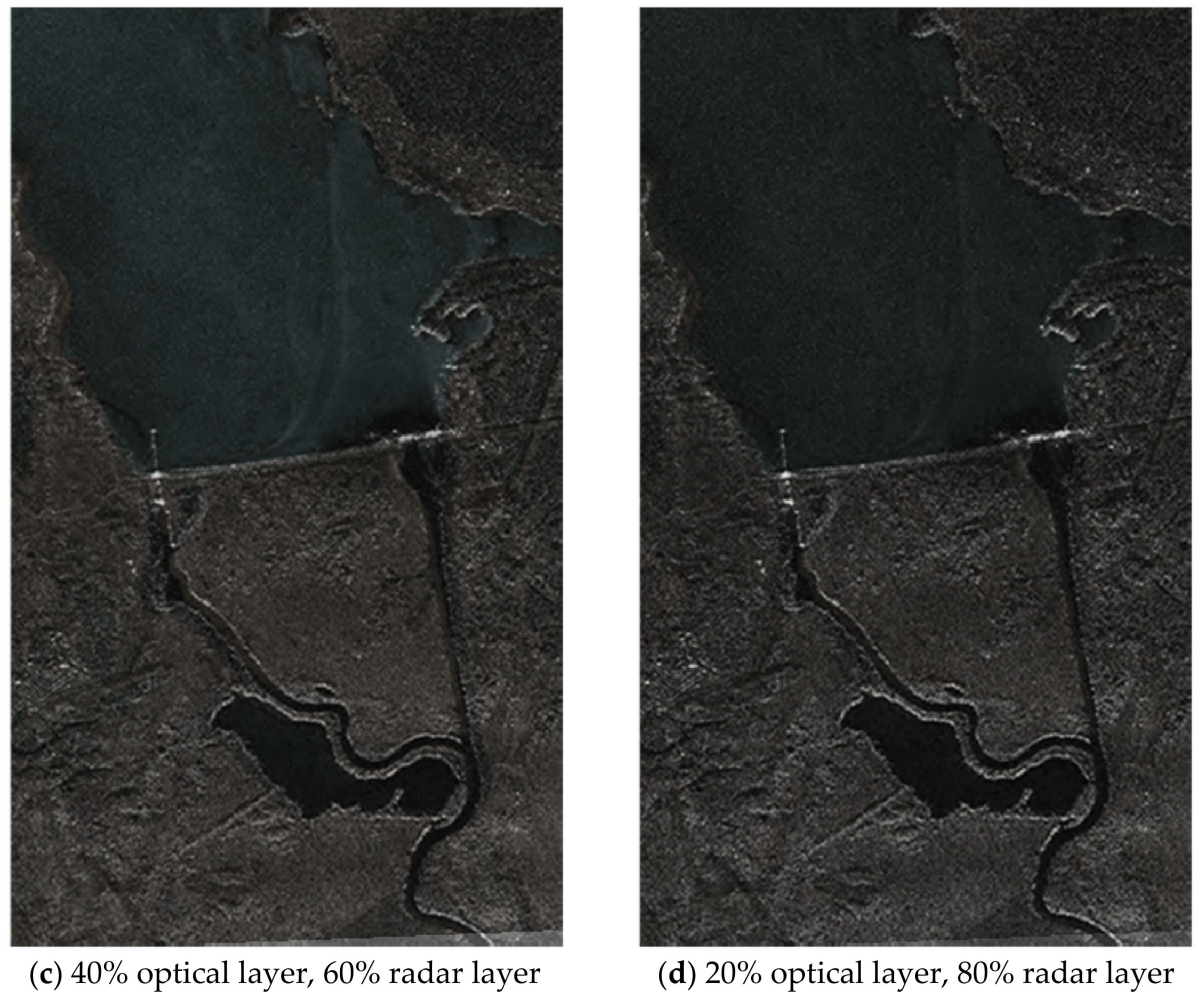
A series of fused images with varying degrees of enhancement of the radar or optical layer.

Enhancement of the radar layer over the optical one is shown in the series of images in Figure 13. In this case (Figure 12) the accuracy of fusion of two different-angle images was ±3–4 pixels when passing to the number of clusters *N*cl = 5 and reaching the autocorrelation lateral lobe level equal to 0.3156.

Thus, in this research, the options for the fusion of images from location sources of different kinds were identified and implemented. The efficiency of the fusion of optical and radar images into a complex one is established. Experiments on the formation of the fused image are presented and the advantages of this approach are described in the implementation of on-board systems for terrain monitoring.

## 5. Conclusions

In this research, a method for construction of a fused image of the land surface for a multi-positional location system for operational monitoring was developed.

The actual choice of a multi-position and multi-sensor system for real-time monitoring of the land surface has been explained. The features of various sources of location information, which are used widely in information integration systems, are considered. It concluded that the fusion of radar and optical information in a single complex image can potentially give a more complete and high-quality representation of the observed areas and objects from the aircraft of the multi-position system.

The workflow of the integration process is given. The main block of this scheme is related to the fusion radar optical images.

To combine heterogeneous multi-angle images, two segmentation methods are used within an intermediate image processing procedure. The pixels of the original images are clustered using the multi-threshold processing method. The execution of the applied pixel clustering algorithm consists of sequential combination of pairs of adjacent clusters on the brightness histogram. The applied pixel clustering algorithm generates a series of partitions, in which the number of clusters gradually reduces from 256 to 1. One or more appropriate partitions can be selected from the generated series. The corresponding filter isolates the borders of the captured areas in the clustered images.

Two new ideas are proposed in this paper: (1) to process a combined image by pixel clustering algorithm to select similar areas in different parts of the two images, that were combines; (2) to select a functional transformation by the contour points selected in the processed pair of clustered images, which is applied to the original images to complex them. The method of complex image formation proposed in this paper can be adapted for testing the algorithms of pixel clustering and complexing of formed images of the frame stream recorded during full-scale tests by heterogeneous location data formation devices.

The implemented method allows us to increase information value, quality and reliability of the finally created combined image of the earth surface.

Experiments on the fusion of optical and radar images of the land surface based on the search for pairs of points of the contours of clustered images are presented. The proposed fusion method is applicable for operational monitoring to ensure environmental control, implementation of search and rescue operations, and other terrain-related aerospace studies. The results of combining heterogeneous (radar and optical) images presented in this work are the basis for providing further research on combining of heterogeneous and different-format information used in modern earth surface monitoring systems.

## Data Availability

All data supporting reported results are public.
